# Supplementary material for: PXL1 and SERKs act as receptor–coreceptor complexes for the CLE19 peptide to regulate pollen development
Source: Nat Commun. 2023 Jun 7;14:3307. doi: 10.1038/s41467-023-39074-4 (PMC10247778; doi:10.1038/s41467-023-39074-4)
Supplement: Supplementary file 1 — Supplementary Information [file 41467_2023_39074_MOESM1_ESM.pdf]

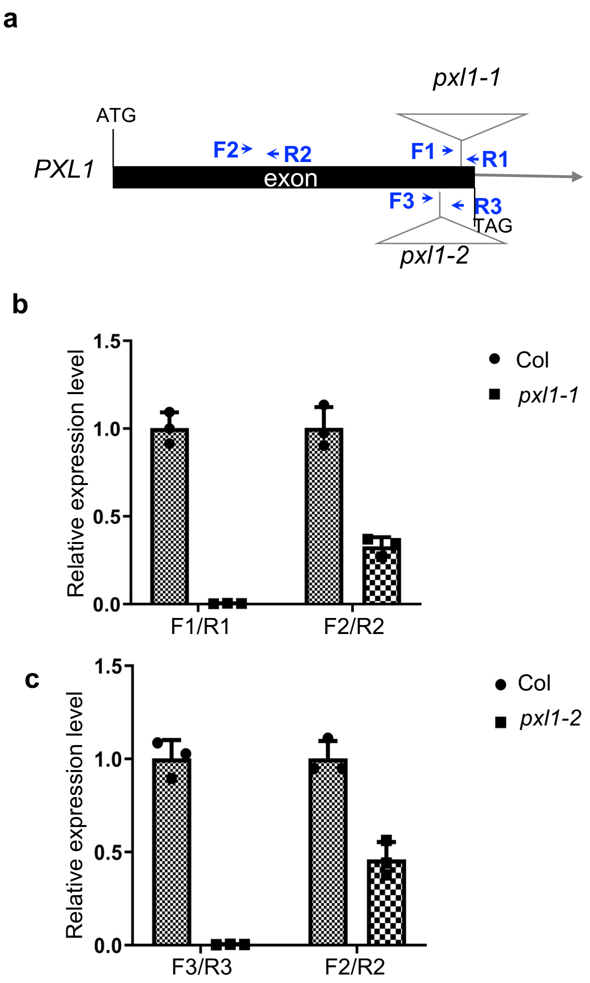

Supplementary Figure 1. Information of the PXL1/2 and PXY T-DNA insertion mutants. (a) Diagrams shows T-DNA insertion sites of *pxl1-1* and *pxl1-2* alleles. Three primer pairs were used to check the relative expression level of PXL1 transcript. F1/R1 primer pair was spanned between *pxl1-1* T-DNA insertion site, F3/R3 was spanned between *pxl1-2* T-DNA insertion site, F2/R2 primer pair was before the T-DNA alleles' insertion site. (b) The relative expression of PXL1 in the inflorescences of WT, *pxl1-1* by two primer pairs (c) The relative expression of PXL1 in the inflorescences of WT, *pxl1-2* by two primer pairs. Data are shown as the mean ± SD. Every dot showed the result for one biological replicate.

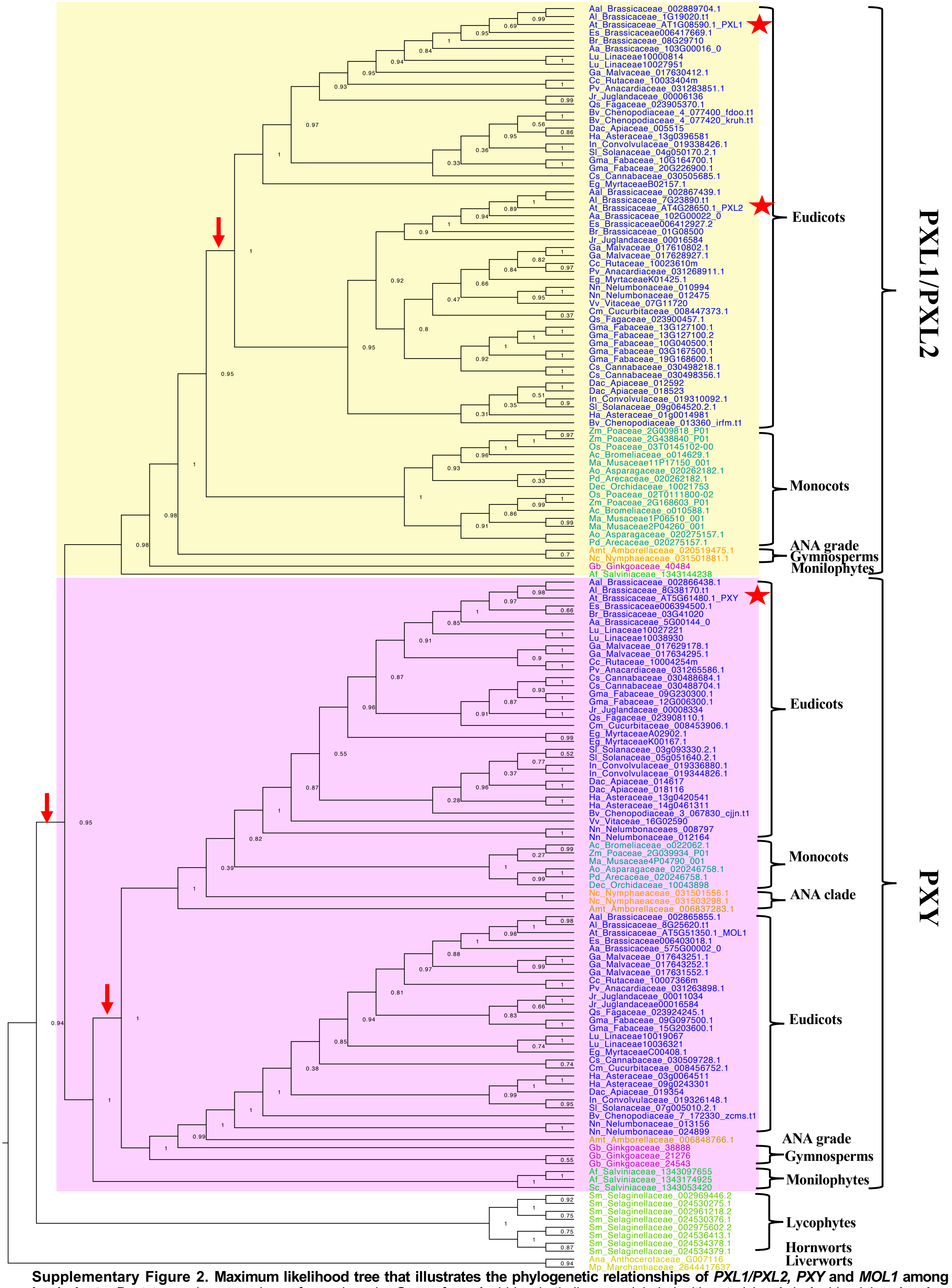

Supplementary Figure 2. Maximum likelihood tree that illustrates the phylogenetic relationships of *PXL1/PXL2, PXY* and *MOL1* among 38 land plants. Bootstrap values are shown for each node. Genes from *Arabidopsis thaliana* are labeled with asterisks. Aal, *Arabis alpine*; Ac, *Ananas comosus* ;Af, *Azolla filiculoides*; Al, *Arabidopsis lyrate*; Amt, *Amborella trichopoda*; Ana, *Anthoceros angustus*; Ao, *Asparagus officinalis*; At, *Arabidopsis thaliana*; Br, *Brassica rapa*; Bv, *Beta vulgaris*; Cc, *Citrus clementina*; Cm, *Cucumis melo*; Cs, *Cannabis sativa*; Dac, *Daucus carota*; Dec, *Dendrobium catenatum*; Eg, *Eucalyptus grandis*; Es, *Eucalyptus grandis*;Ga, *Gossypium arboretum*; Gb, *Ginkgo biloba*; Gma, *Glycine max*; Ha, *Helianthus annuus*; In, *Ipomoea nil*; Jr, *Juglans regia*; Lu, *Linum usitatissimum*; Ma, *Musa acuminata*; Ma, *Musa acuminata*; Mp, *Marchantia polymorpha*; Nc, *Nymphaea colorata*; Nn, *Nelumbo nucifera*; Os, *Oryza sativa*; Pd, *Phoenix dactylifera*; Pv, *Pistacia vera*; Qs, *Quercus suber*; Sc, *Salvinia cucullate*; Sl, *Solanum lycopersicum*; Sm, *Selaginella moellendorffii*; Vv, *Vitis vinifera*; Zm, *Zea mays*.

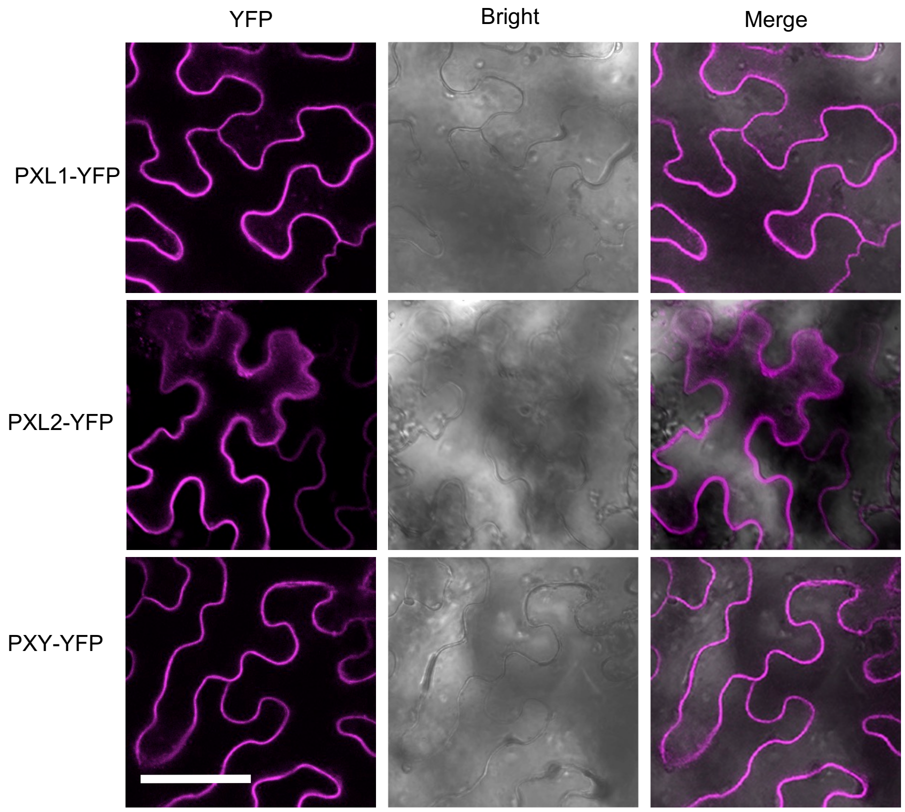

Supplementary Figure 3. Subcellular localization of PXL1-YFP, PXL2-YFP, and PXY-YFP in Nicotiana benthamiana leaf transient expression system. From left to right are images from the YFP channel (magenta), the bright field channel (black and white), and merged images. Bar = 50 μm. Three times experiments were repeated with similar results.

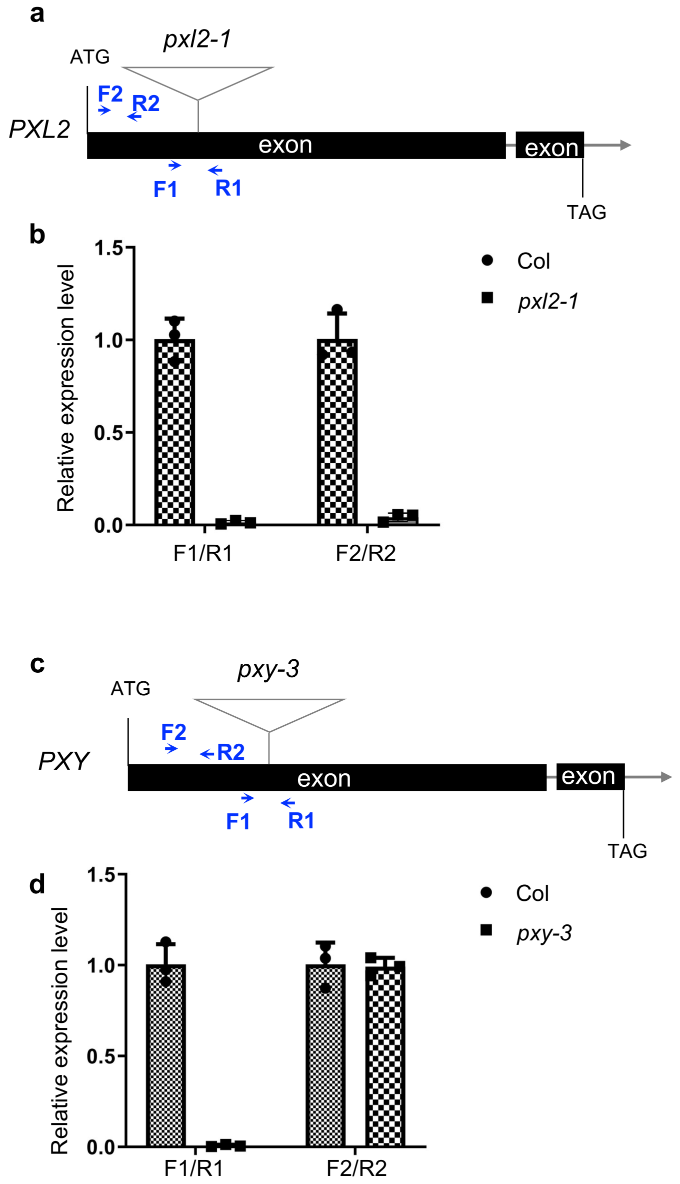

Supplementary Figure 4. Information of the PXL1/2 and PXY T-DNA insertion mutants. (a) Diagrams shows T-DNA insertion sites of pxl2-1 allele. Two primer pairs were used to check the relative expression level of PXL2 transcript. F1/R1 primer pair was spanned between pxl2-1 T-DNA insertion site, F2/R2 primer pair was before the two T-DNA allele's insertion sites. (b) The relative expression of PXL2 in the inflorescences of WT, *pxl2-1* by two primer pairs. (c) Diagrams shows T-DNA insertion sites of *pxy-3* allele. Two primer pairs were used to check the relative expression level of PXY transcript. F1/R1 primer pair was spanned between *pxl2-1* T-DNA insertion site, F2/R2 primer pair was before the two T-DNA allele's insertion sites. (d) The relative expression of PXY in the inflorescences of WT, *pxy-3* by two primer pairs. Data in (b) and (d) are shown as the mean ± SD. Every dot showed the result for one biological replicate. The expression of ACTIN was used as the internal control.

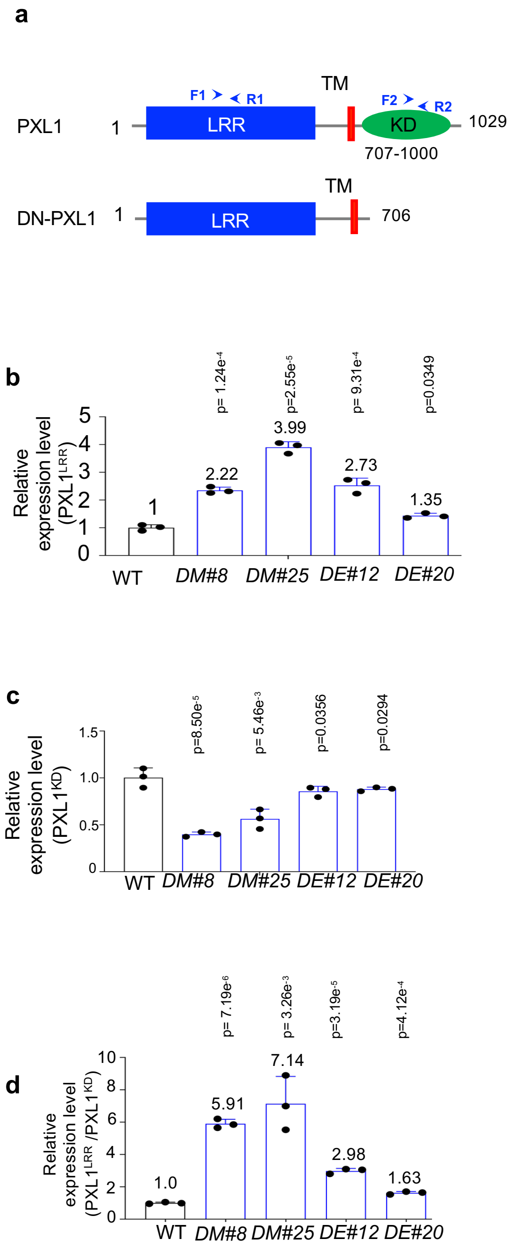

Supplementary Figure 5. Q-RT PCR analysis of different DN-PXL1 transgenic plants. (a) Schematic diagram shows the domain structure of PXL1 protein. PXL1 contains a phosphatase catalytic domain (KD, green), a transmembrane domain (TM, red) and the predicted LRR motifs (blue). (b) F1/R1 primer pair on the LRR domain of PXL1 was used to check the expression level of $PXL1^{LRR}$ transcript. (c) F2/R2 primer pair on the KD domain of PXL1 was used to check the expression level of $PXL1^{KD}$ transcript. Data in (b-c), every dot showed the result for one biological replicate. Data are shown as the mean ± SD of three replicate. P values were calculated by student t-Test, two sided. (d) the ratio of $PXL1^{LRR}/PXL1^{KD}$ expression level. The expression of ACTIN was used as the internal control. Three independent biological replicates were performed. Every dot showed the result for one biological replicate. Data are shown as the mean ± SD. P values were calculated by student t-Test, two sided.

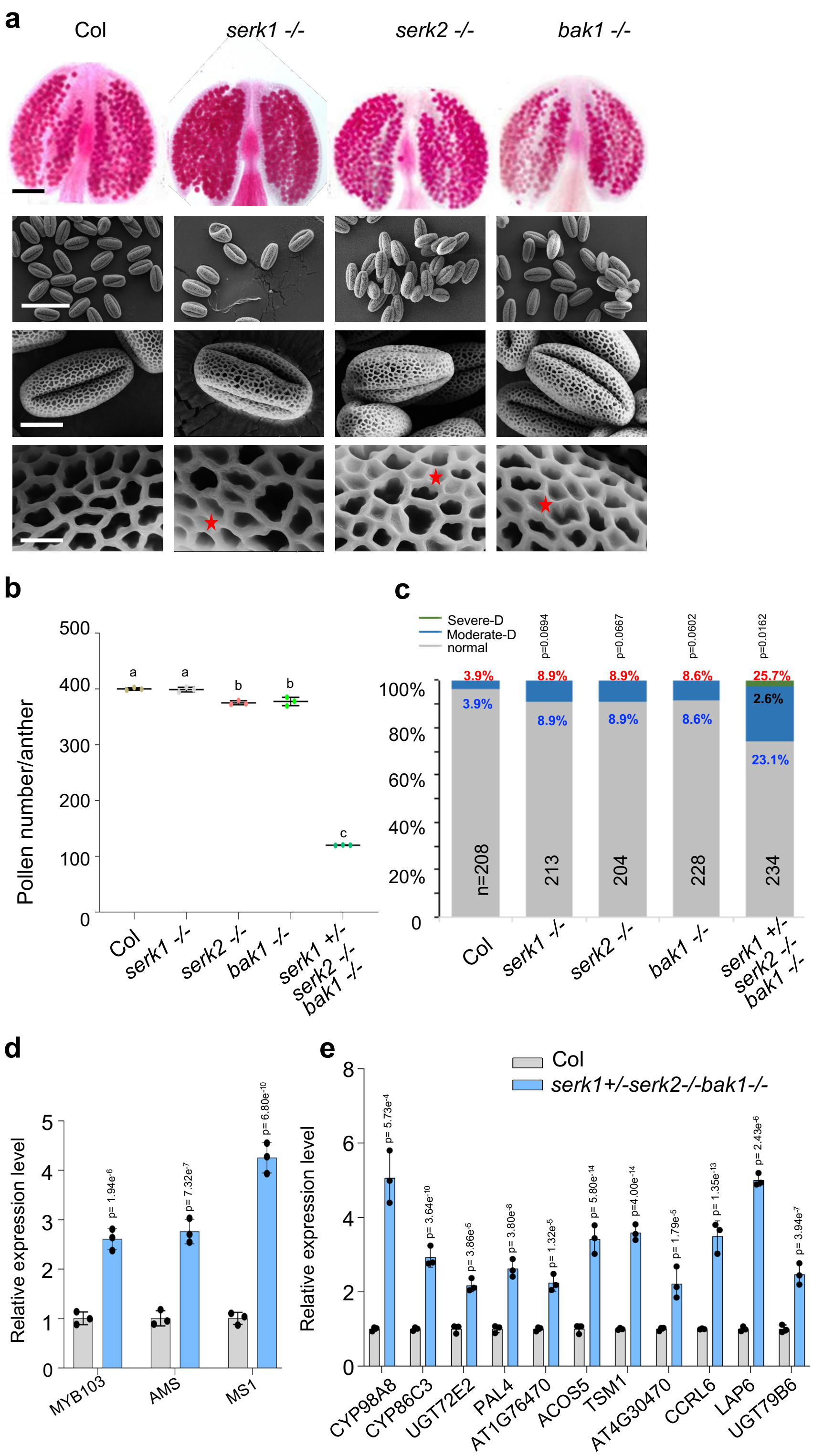

Supplementary Figure 6. SERKs play role in pollen exine formation. (a) Phenotypic analyses of the serk1-/-, serk2-/- and bak1-/- single mutants, including alexander staining and SEM observation. bar=100 μm for anther, 50 μm for the first row of SEM images, 10 μm for the second row of SEM images, 2 μm for the bottom row of SEM images, respectively. The red star indicated the pollen exine defect. The red stars indicate the pollen exine defect. (b) Quantification of pollen amounts per anther in the indicated genotypes. Three independent biological replicates were performed, and ten anthers were used for analysis for each replicate. The data is shown as the mean ± SD, each dot shows the average value for one biological replicate. Different letters represent significant difference between each other, $P < 0.05$, one-way ANOVA with Tukey multiple comparison test. Exact P values are 0.989 for *serk1-/-* vs Col, 1.33e-4 for *serk2-/-* vs Col, 2.96e-4 for *bak1-/-* vs Col, 3.11e-9 for *serk1+/- serk2-/- bak1-/-* vs Col. (c) Statistical analyses show the portion of pollen with normal, moderate-D and severe-D exine defects in each of the Col-0, *serk1-/-*, *serk2-/-,* and *bak1-/-* anther. P values were calculated by chi-square. Exact P values are 0.0694 for *serk1-/-* vs Col, 0.0667 for *serk2-/-* vs Col, 0.0602 for *bak1-/-* vs Col, 0.0162 for *serk1+/- serk2-/- bak1-/-* vs Col. (d) qRT–PCR results showed the relative expression of the DYT1, AMS, MYB103, MS1 in WT and *serk1-/-*, *serk2-/-,* and *bak1-/-.* (e) qRT–PCR results showed the relative expression of the CYP98A8, CYP86C3, AT5G55320, UGT72E2, PAL4, AT1G76470, and ACOS5 in WT and *serk1-/-, serk2-/-,* and *bak1-/-,* The expression of ACTIN was used as the internal control. The data in (d-e) is shown as the mean ± SD of three biological replicates. Each dot showed the result for one biological replicate. P values were calculated by student t-Test, two-sided.

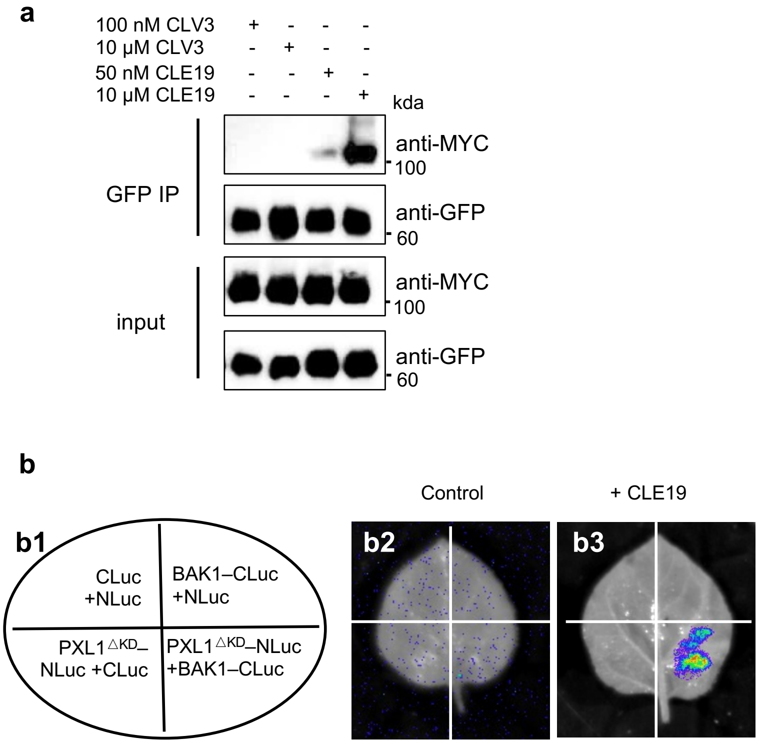

Supplementary Figure 7. CLE19 promotes the interaction between PXL1 and BAK1. (a) In vivo co-immunoprecipitation assay showing the interaction between PXL1 and BAK1 with or without CLE19 treatment. $PXL1^{\Delta KD}$, PXL1-with kinase domain deletion; $BAK1^{\Delta KD}$, BAK1-with kinase domain deletion. GFP-tagged $BAK1^{\Delta KD}$ and MYC-tagged $PXL1^{\Delta KD}$ were co-expressed in Nicotiana benthamiana. Three times experiments were repeated with similar results. (b) Split luciferase assays show the interaction between PXL1 and BAK1 with or without 20 μM CLE19 treatment. b1 shows the model of the infiltration protein pairs, b2 and b3 show the results of control and 20 μm CLE19 treatment respectively.

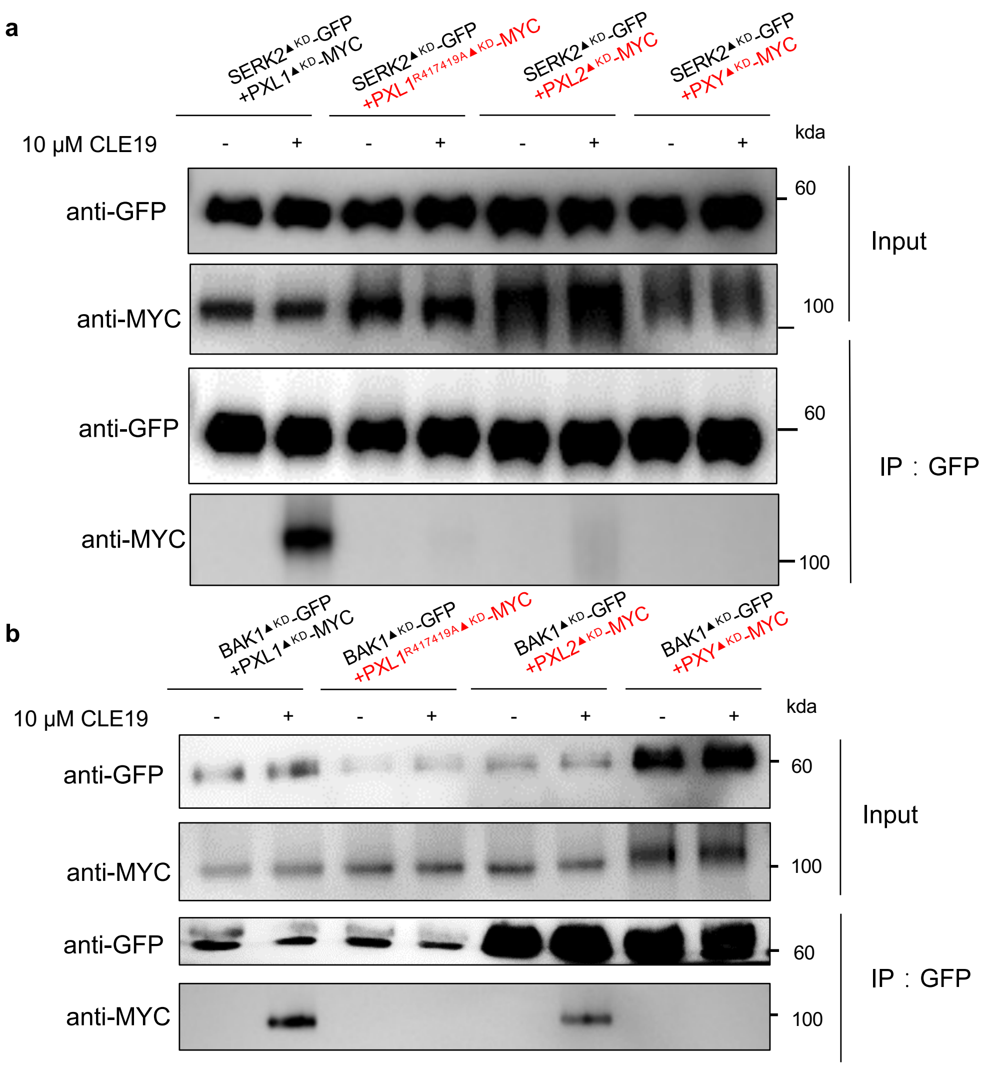

Supplementary Figure 8. In vivo co-immunoprecipitation showed the interaction between PXL1/PXL2/PXY with SERK2 and BAK1 with or without CLE19 treatment. (a) SERK2KD -GFP protein was co-expressed with $PXL1^{\Delta KD}$/$PXL1^{\Delta KD\text{-}R417419A}$/$PXL2^{\Delta KD}$/$PXY^{\Delta KD}$-MYC in Nicotiana benthamiana. Anti-MYC and anti-GFP was used to indicate the protein level, GFP beads were used to perform the IP process. (b) BAK1 $^{\Delta KD}$-GFP protein was co-expressed with $PXL1^{\Delta KD}$/$PXL1^{\Delta KD\text{-}R417419A}$/$PXL2^{\Delta KD}$ /$PXY^{\Delta KD}$ -MYC in Nicotiana benthamiana. Anti-MYC and anti-GFP was used to indicate the protein level, GFP beads were used to perform the IP process. Three times experiments were repeated with similar results for (a-b).

Supplementary Table 1. CLE peptide used in this study

Peptide	Sequence (from N terminus to C terminus)
CLV3	RTV(Hyp)SG(Hyp)DPLHH
CLE3	RLSPGG(Hyp)DPRHH
CLE6	RVSPGG(Hyp)DPQHH
CLE8	RRVPTG(Hyp)NPLHH
CLE9	RLV(Hyp)SG(Hyp)NPLHN
CLE11	RVVPSG(Hyp)NPLHH
CLE12	RRVPSG(Hyp)NPLHH
CLE14	RLVPKG(Hyp)NPLHN
CLE16	RLVHTG(Hyp)NPLHN
CLE18	RQIPTG(Hyp)DPLHN
CLE19	RVIPTG(Hyp)NPLHN
$CLE19_{G6T}$	RVIPTTNPLHN
CLE20	RKVKTGSNPLHN
CLE25	RKVPNG(Hyp)DPIHN
CLE40	RQVPTGSDPLHH
TDIF	HEV(Hyp)SG(Hyp)NPISN
CLE43	RRIPSS(Hyp)DRLHN
CLE45	RRVRRGSDPIHN
CLE46	HKHPSG(Hyp)NPTGN

Supplementary Table 2. The 41 land plants used for analysis of PXL1 homologue genes

source	ID	species	family	orders	represent clade
Phytozome	At	Arabidopsis_thaliana	Brassicaceae	Brassicales	Eudicots
NCBI_refseq	Aa	Aethionema arabicum	Brassicaceae	Brassicales	Eudicots
Phytozome	Al	Arabidopsis lyrata	Brassicaceae	Brassicales	Eudicots
NCBI_refseq	Aal	Arabis alpina	Brassicaceae	Brassicales	Eudicots
NCBI_refseq	Br	Brassica rapa	Brassicaceae	Brassicales	Eudicots
Phytozome	Es	Eutrema salsugineum	Brassicaceae	Brassicales	Eudicots
NCBI_refseq	Ga	Gossypium_arboreum	Malvaceae	Malvales	Eudicots
Phytozome	Lu	Linum_usitatissimum	Linaceae	Malpighiales	Eudicots
NCBI_refseq	Cs	Cannabis_sativa	Cannabaceae	Rosales	Eudicots
NCBI_refseq	Cm	Cucumis_melo	Cucurbitaceae	Cucurbitales	Eudicots
NCBI_refseq	Qs	Quercus_suber	Fagaceae	Fagales	Eudicots
NCBI_refseq	Jr	Juglans_regia	Juglandaceae	Fagales	Eudicots
Phytozome	Eg	Eucalyptus_grandis	Myrtaceae	Myrtales	Eudicots
Phytozome	Cc	Citrus_clementina	Rutaceae	Spindales	Eudicots
NCBI_refseq	Pv	Pistacia_vera	Anacardiaceae	Sapindales	Eudicots
NCBI_refseq	Gma	Glycine_max	Fabaceae	Fabales	Eudicots
Phytozome	Vv	Vitis_vinifera	Vitaceae	Vitales	Eudicots
NCBI_refseq	Dac	Daucus_carota	Apiaceae	Apiales	Eudicots
NCBI_refseq	Ha	Helianthus_annuus	Asteraceae	Asterales	Eudicots
NCBI_refseq	In	Ipomoea_nil	Convolvulaceae	Solanales	Eudicots
NCBI_refseq	Sl	Solanum_lycopersicum	Solanaceae	Solanales	Eudicots
NCBI_refseq	Bv	Beta_vulgaris	Chenopodiaceae	Caryophyllales	Eudicots
NCBI_refseq	Nn	Nelumbo_nucifera	Nelumbonaceae	Proteales	Eudicots
NCBI_refseq	Pd	Phoenix_dactylifera	Arecaceae	Arecales	Monocots
NCBI_refseq	Ao	Asparagus_officinalis	Asparagaceae	Asparagales	Monocots
NCBI_refseq	Ac	Ananas_comosus	Bromeliaceae	Poales	Monocots
NCBI_refseq	Ma	Musa_acuminata	Musaceae	Zingiberales	Monocots
NCBI_refseq	Dec	Dendrobium_catenatum	Orchidaceae	Asparagales	Monocots
NCBI_refseq	Os	Oryza_sativa	Poaceae	Poales	Monocots
NCBI_refseq	Zm	Zea_mays	Poaceae	Poales	Monocots
NCBI_refseq	Amt	Amborella_trichopoda	Amborellaceae	Amborellales	ANA clade
NCBI_refseq	Nc	Nymphaea_colorata	Nymphaeaceae	Nymphaeales	ANA clade
NCBI_refseq	Gm	Gnetum montanum	Gnetaceae	Gnetales	Gymnosperms
NCBI_refseq	Pt	Pinus taeda	Pinaceae	Pinales	Gymnosperms
NCBI_refseq	Gb	Ginkgo biloba	Ginkgoaceae	Ginkgoales	Gymnosperms
NCBI_refseq	Sm	Selaginella moellendorffii	Selaginellaceae	Selaginellales	Lycophytes
NCBI_refseq	Af	Azolla filiculoides	Salviniaceae	Salviniales	Monilophytes
NCBI_refseq	Sc	Salvinia cucullata	Salviniaceae	Salviniales	Monilophytes
NCBI_refseq	Pp	Physcomitrella patens	Funariaceae	Funariales	Mosses
NCBI_refseq	Ana	Anthoceros angustus	Anthocerotaceae	Anthocerotales	Hornworts
NCBI_refseq	Mp	Marchantia polymorpha	Marchantiaceae	Marchantiales	Liverworts

Supplemental Table 3. Primers used in this work

Primer name	Primer (5'-3')	Function
pxl1-1-FP	AATCGATGGTCTATCCTTCGG	For genotyping of *pxl1-1*
pxl1-1-RP	TATGCGGTGGAGTTCTACCAC	For genotyping of *pxl1-1*
pxl1-2-FP	CAGGAGCATCTCTTCGATCAC	For genotyping of *pxl1-2*
pxl1-2-RP	GCCATAGACCCAAAAGACCTC	For genotyping of *pxl1-2*
pxl2-1-FP	ACCTCTATGCCACACACCAAG	For genotyping of *pxl2-1*
pxl2-1-RP	CAAGCTCTGACGGAATCTCAC	For genotyping of *pxl2-1*
pxy-3-FP	CCCCACACAAAAACCATAATG	For genotyping of *pxy-3*
pxy-3-RP	AAAAATCGAGAAGCTTGAGGG	For genotyping of *pxy-3*
RT-*pxl1-1*-F1	CAGAAGTCATGAGACGGCCA	For real-time PCR of *pxl1-1* mutant
RT-*pxl1-1*-R1	TTCCATTGGGCATGTACTCGT	For real-time PCR of *pxl1-1* mutant
RT-*pxl1-1*-F2	AGGAGGTTACTTCGAGGGCT	For real-time PCR of *pxl1-1* mutant
RT-*pxl1-1*-R2	GCGCGTCAATTTCCCAAACT	For real-time PCR of *pxl1-1* mutant
RT-*pxl1-2*-F3	AATGGCCATGGAGACTCGTG	For real-time PCR of *pxl1-2* mutant
RT-*pxl1-2*-R3	CCGTCTCATGACTTCTGCTTTG	For real-time PCR of *pxl1-2* mutant
RT-*pxl2-1*-F1	GGTGCAACTCAAATGGCAAT	For real-time PCR of *pxl2-1* mutant
RT-*pxl2-1*-R1	ACTCGAATCCGTTGCATGATAT	For real-time PCR of *pxl2-1* mutant
RT-*pxl2-1*-F2	CCATCGACAATGTCAACGAGC	For real-time PCR of *pxl2-1* mutant
RT-*pxl2-1*-R2	ACAGTGATCGCTGGTGTCTG	For real-time PCR of *pxl2-1* mutant

Primer name	Primer (5'-3')	Function
RT-*pxy-3*-F1	TGCGTTCAGCAACAACTTCG	For real-time PCR of *pxy-3* mutant
RT-*pxy-3*-R1	CCACCGTAAGCTGCTGGAAT	For real-time PCR of *pxy-3* mutant
RT-*pxy-3*-F2	TCGACATCAGCCGTAACTCG	For real-time PCR of *pxy-3* mutant
RT-*pxy-3*-R2	CGAAGTTGTTGCTGAACGCA	For real-time PCR of *pxy-3* mutant
RT-PXL1^{LRR} -FP	CACACGTCAATGCCTCAAGC	For real-time PCR of $PXL1^{LRR}$ transcripts
RT-PXL1^{LRR} -RP	AAGAAGAAGGCACTGAGCCC	For real-time PCR of $PXL1^{LRR}$ transcripts
RT-PXL1^{KD} -FP	CAGAAGTCATGAGACGGCCA	For real-time PCR of full length of *PXL1* transcripts
RT-PXL1^{KD} -RP	TTCCATTGGGCATGTACTCGT	For real-time PCR of full length of *PXL1* transcripts
SALK_071511-LP	ACTGAAGGAAGAGCGAACTCC	For genotyping of *serk1*
SALK_071511-RP	TTGGACCAGATAACTCAACGG	For genotyping of *serk1*
SALK_058020-LP	AGTGAAGAGCGAGAAGGAACC	For genotyping of *serk2*
SALK_058020-RP	AAGGCTTAGGCTTTTGTTTGG	For genotyping of *serk2*
SALK_116202-LP	CATGACATCATCATCATTCGC	For genotyping of *bak1*
SALK_116202-RP	ATTTTGCAGTTTTGCCAACAC	For genotyping of *bak1*
LBb1.3	ATTTTGCCGATTTCGGAAC	For genotyping border primer
RT-*DYT1*-LP1	GAGAGACGTAGAAGAGAGAAGC	For real-time PCR of *DYT1* transcripts

Primer name	Primer (5'-3')	Function
RT-*DYT1*-RP1	AGGAGCTTCTTCCATTTCATGA	For real-time PCR of *DYT1* transcripts
RT-*AMS*-LP1	TATGAATGAGAACGGTAGGGTG	For real-time PCR of *AMS* transcripts
RT-*AMS*-RP1	CTCATTCTGCAACTCCTTAACG	For real-time PCR of *AMS* transcripts
RT-*MYB103*-LP1	AACTACTGTGACATTGAATGCG	For real-time PCR of *MYB103* transcripts
RT-*MYB103*-RP1	TAGGAGAATCAGTGAAACCGTC	For real-time PCR of *MYB103* transcripts
RT-*MS1*-LP1	CAGCTCGAGATTCAACGAAATC	For real-time PCR of *MS1* transcripts
RT-*MS1*-RP1	TCATTTGATTTCCCCAACCAAC	For real-time PCR of *MS1* transcripts
RT-*PXL1*-FP	AAACTCGACGGACCCATTCC	For real-time PCR of *PXL1* transcripts
RT-*PXL1*-RP	CTCCCTGGATTTCGGCCTTT	For real-time PCR of *PXL1* transcripts
RT-*CLE19*-LP1	GCGGAGTTTGCTGATGAATAAT	For real-time PCR of *CLE19* transcripts
RT-*CLE19*-RP1	TTACCTGTTGTGGAGTGGATTT	For real-time PCR of *CLE19* transcripts
RT-*CYP98A8*-FP	GCATCGAAAATTTCAGGTCCTT	For real-time PCR of *CYP98A8* transcripts
RT-*CYP98A8*-RP	TCCCCTGAATTGCTCATTAAGT	For real-time PCR of *CYP98A8* transcripts
RT-*CYP86C3*-FP	AAAGAACGGTTCAATGATCTGC	For real-time PCR of *CYP86C3* transcripts
RT-*CYP86C3*-RP	CTGGTTGAATGCATTTCGGTAA	For real-time PCR of *CYP86C3* transcripts
RT-AT5G55320-FP	TTTGTCCACGTATGTTTTTCCC	For real-time PCR of AT5G55320 transcripts
RT- AT5G55320-RP	GTTCGTGAAAATGTGGCTCTAG	For real-time PCR of AT5G55320 transcripts

Primer name	Primer (5’-3’)	Function
RT-*UGT72E2*-FP	TTCGTGTTGTAGCGAGTATGTC	For real-time PCR of *UGT72E2* transcripts
RT-*UGT72E2*-RP	GACCACGAAACCTCTATCACTA	For real-time PCR of *UGT72E2* transcripts
RT-*PAL4*-FP	CCCTAGTCTTGATTACGGGTTT	For real-time PCR of *PAL4* transcripts
RT-*PAL4*-RP	ATTAACGTCTTGGTTATGCTGC	For real-time PCR of *PAL4* transcripts
RT-AT1G76470-FP	GCTCTGTCCGTCTGTCATTATA	For real-time PCR of AT1G76470 transcripts
RT-AT1G76470-RP	GCACATCCACAAGATACAGTTC	For real-time PCR of AT1G76470 transcripts
RT-*ACOS5*-FP	AAGTTGTTGCTATGAGTCGGTA	For real-time PCR of *ACOS5* transcripts
RT-*ACOS5*-RP	TATAATCGGCGGAACTATAGGC	For real-time PCR of *ACOS5* transcripts
RT-*TSM1*-FP	TTACTCACTTTTCACCGTAGCT	For real-time PCR of *TSM1* transcripts
RT-*TSM1*-RP	TCTTGTTTCTCCCCGTTCAATA	For real-time PCR of *TSM1* transcripts
RT-AT4G30470-FP	GTTAAGTTTCTAGCGGACGTTC	For real-time PCR of AT4G30470 transcripts
RT-AT4G30470-RP	CTTCTGTGTTGACGATTTGGTT	For real-time PCR of AT4G30470 transcripts
RT-*CCRL6*-FP	GGACCAAAACCAACAAGTACTC	For real-time PCR of *PXL1* transcripts
RT-*CCRL6*-RP	CTTCCATAGCCAATACATGTGC	For real-time PCR of *PXL1* transcripts
RT-*LAP6*-FP	GTCGATGGAGTTCAATGACATG	For real-time PCR of *LAP6* transcripts

Primer name	Primer (5'-3')	Function
RT-*LAP6*-RP	CACATACAGTATCGTGTTGCTG	For real-time PCR of *LAP6* transcripts
RT-*UGT79B6*-FP	TTTCTTCGATTTTGCTCACTGG	For real-time PCR of *UGT79B6* transcripts
RT-*UGT79B6*-RP	CATGCTGCAGAAATCGTTATGA	For real-time PCR of *UGT79B6* transcripts
Actin-F	ATCGGTGGTTCCATTCTTGCTTC	For real-time PCR of *Actin* transcripts
Actin-R	TGGACCTGCCTCATCATACTCG	For real-time PCR of *Actin* transcripts
CLE19-attb-F	GGGGACAAGTTTGTACAAAAAAGCAGGCTTCATGAAGATAAAGGGTTTGATGATAT	For *35S::CLE19-FLAG* construct
CLE19-attb-R	GGGACCACTTTGTACAAGAAAGCTGGGTCCCTGTTGTGGAGTGGATTTGG	For *35S::CLE19-FLAG* construct
pPXL1-attb-F	GGGGACAAGTTTGTACAAAAAAGCAGGCTTCACTTAATGCTACTTTGTTCTTCACG	For *DN-PXL1* construct
pPXL1-R	NNNNGGATCCTTTTCTCCTCTATTTCTTCACTTTT	For *DN-PXL1* construct
DN-PXL1-F	NNNNGGATCCATGGCGATCCCTCGACTTTT	For *DN-PXL1* construct
DN-PXL1-attb-R	GGGGACCACTTTGTACAAGAAAGCTGGGT C ATGTGATAAGATGTCTCCTGCAGTG	For *DN-PXL1* construct
SERK1$^{\triangle KD}$ -F	NNNNGGTACCATGGAGTCGAGTTATGTGGTG	For SERK1$^{\triangle KD}$ construct

Primer name	Primer (5’-3’)	Function
SERK1$^{\triangle KD}$ -F	NNNNGGTACCATGGAGTCGAGTTATGTGGTG	For SERK1$^{\triangle KD}$ construct
SERK1$^{\triangle KD}$-R	NNNNGTCGACAGGGACATCGAAGAAAATATCTAG	For SERK1$^{\triangle KD}$ construct
SERK2$^{\triangle KD}$ -F	NNNNGGTACCATGGGGAGAAAAAAGTTTGAAG	For SERK2$^{\triangle KD}$ construct
SERK2$^{\triangle KD}$ -R	NNNNGTCGACAGGAACATCAAAGAAGAATTC	For SERK2$^{\triangle KD}$ construct
BAK1$^{\triangle KD}$-F	NNNNGGTACCATGGAACGAAGATTAATGATC	For BAK1$^{\triangle KD}$ construct
BAK1$^{\triangle KD}$-R	NNNNGTCGACTGGTACATCAAAGAAGTGGTCCTG	For BAK1$^{\triangle KD}$ construct
PXL1$^{\triangle KD}$-F	NNNNGGATCCATGGCGATCCCTCGACTTTT	For PXL1$^{\triangle KD}$ construct
PXL1$^{\triangle KD}$-R	NNNNGTCGACATGTGATAAGATGTCTCCTGCAGTG	For PXL1$^{\triangle KD}$ construct
PXL1$^{\triangle KD\text{-}R417419A}$ -F	TTGCTGTTGCAATCCAGAAAAATCACATTTCCGG	For PXL1$^{\triangle KD\text{-}R417419A}$ construct
PXL1$^{\triangle KD\text{-}R417419A}$ -R	CTAAAGTCGGACATGAGAAGATTTC	For PXL1$^{\triangle KD\text{-}R417419A}$ construct

Primer name	Primer (5'-3')	Function
PXL2$^{\triangle KD}$-F	NNNNGGATCCATGAAGATGAAGATAATAGTT	For PXL2$^{\triangle KD}$ construct
PXL2$^{\triangle KD}$-R	NNNNGTCGACACATGCCAAGATATCGGAAGC	For PXL2$^{\triangle KD}$ construct
PXY$^{\triangle KD}$-F	NNNNGGATCCATGAAAAAGAAGAACATTTCTCC	For PXY$^{\triangle KD}$ construct
PXY$^{\triangle KD}$-R	NNNNGTCGACGAGACACTCAACCACATCATC	For PXY$^{\triangle KD}$ construct
