# Supplementary material for: PXL1 and SERKs act as receptor–coreceptor complexes for the CLE19 peptide to regulate pollen development
Source: Nat Commun. 2023 Jun 7;14:3307. doi: 10.1038/s41467-023-39074-4 (PMC10247778; doi:10.1038/s41467-023-39074-4)
Supplement: Supplementary file 3 — reporting summary [file 41467_2023_39074_MOESM3_ESM.pdf]

nature portfolio

Corresponding author(s): Fang Chang

Last updated by author(s): May 8, 2023

Reporting Summary

Nature Portfolio wishes to improve the reproducibility of the work that we publish. This form provides structure for consistency and transparency in reporting. For further information on Nature Portfolio policies, see our Editorial Policies and the Editorial Policy Checklist.

Statistics

For all statistical analyses, confirm that the following items are present in the figure legend, table legend, main text, or Methods section.

n/a	Confirmed	
☐	☒	The exact sample size (*n*) for each experimental group/condition, given as a discrete number and unit of measurement
☐	☒	A statement on whether measurements were taken from distinct samples or whether the same sample was measured repeatedly
☐	☒	The statistical test(s) used AND whether they are one- or two-sided<br>*Only common tests should be described solely by name; describe more complex techniques in the Methods section.*
☒	☐	A description of all covariates tested
☐	☒	A description of any assumptions or corrections, such as tests of normality and adjustment for multiple comparisons
☐	☒	A full description of the statistical parameters including central tendency (e.g. means) or other basic estimates (e.g. regression coefficient) AND variation (e.g. standard deviation) or associated estimates of uncertainty (e.g. confidence intervals)
☐	☒	For null hypothesis testing, the test statistic (e.g. *F*, *t*, *r*) with confidence intervals, effect sizes, degrees of freedom and *P* value noted<br>*Give P values as exact values whenever suitable.*
☒	☐	For Bayesian analysis, information on the choice of priors and Markov chain Monte Carlo settings
☒	☐	For hierarchical and complex designs, identification of the appropriate level for tests and full reporting of outcomes
☒	☐	Estimates of effect sizes (e.g. Cohen's *d*, Pearson's *r*), indicating how they were calculated

Our web collection on statistics for biologists contains articles on many of the points above.

Software and code

Policy information about availability of computer code

Data collection	gel filtration data was collected by Superdex 200 (GE Healthcare); ITC assay was collected by MicroCal ITC200 (GE Healthcare); qRT-PCR data was collected by a CFX96 Touch Real-Time PCR detection system (Bio-Rad).
Data analysis	Statistical analysis: GraphPad Prism 7<br>Image analysis: Image J 1.8.0 and Quantity One v462<br>ITC data: MicroCal Origin 7.0

For manuscripts utilizing custom algorithms or software that are central to the research but not yet described in published literature, software must be made available to editors and reviewers. We strongly encourage code deposition in a community repository (e.g. GitHub). See the Nature Portfolio guidelines for submitting code & software for further information.

Data

Policy information about availability of data

All manuscripts must include a data availability statement. This statement should provide the following information, where applicable:

- Accession codes, unique identifiers, or web links for publicly available datasets
- A description of any restrictions on data availability
- For clinical datasets or third party data, please ensure that the statement adheres to our policy

The data supporting the findings in this study are available and described within the paper and its supplemental information. Source Data (gels and graphs) for Figs. 1-5 and supplementary Figs. 1, 5, 4-8 are provided as a separate Source Data file.

Field-specific reporting

Please select the one below that is the best fit for your research. If you are not sure, read the appropriate sections before making your selection.

☒ Life sciences ☐ Behavioural & social sciences ☐ Ecological, evolutionary & environmental sciences

For a reference copy of the document with all sections, see nature.com/documents/nr-reporting-summary-flat.pdf

Life sciences study design

All studies must disclose on these points even when the disclosure is negative.

Sample size	Sample size was determined by previous publications. These were clearly indicated in each figure legends or methods.
Data exclusions	No data was excluded.
Replication	All attempts of replication by different people within the group were successful.
Randomization	The samples were picked to do biochemistry assay is randomized; seedlings with similar growth condition were used for root phenotypic analyses.
Blinding	The key phenotype analysis was confirmed by double blind. We did not do blinding assay to biochemistry data because the loading of gel need a known sample order.

Reporting for specific materials, systems and methods

We require information from authors about some types of materials, experimental systems and methods used in many studies. Here, indicate whether each material, system or method listed is relevant to your study. If you are not sure if a list item applies to your research, read the appropriate section before selecting a response.

Materials & experimental systems

n/a	Involved in the study	
☐	☒	Antibodies
☐	☒	Eukaryotic cell lines
☒	☐	Palaeontology and archaeology
☒	☐	Animals and other organisms
☒	☐	Clinical data
☒	☐	Dual use research of concern

Methods

n/a	Involved in the study	
☒	☐	ChIP-seq
☒	☐	Flow cytometry
☒	☐	MRI-based neuroimaging

Antibodies

Antibodies used	anti-MYC (GNI4110-MC), Shang hai Rui yuan biotech, 1:2500 dilution;<br>anti-GFP (M20004), Abmart, 1:2500 dilution;<br>anti-FLAG(GNI4110-FG), Shang hai Rui yuan biotech, 1:2500 dilution;<br>anti-p (PP2551), ECM, 1:1000 dilution;<br>anti-HSP (M20041), Abmart, 1:2500 dilution<br>Goat anti-mouse IgG (H+L)-HRP Conjugated (115-035-003), Jakson, 1:2000 dilution.<br>Goat anti-rabbit IgG-HRP Conjugated (M21002), Abmart, 1:2000 dilution.
Validation	Validation statements of commercial primary antibodies are available from manufactures: anti-MYC (http://fulaibio.com/index.php?m=home&c=View&a=index&aid=554),<br>anti-GFP (http://www.ab-mart.com.cn/page.aspx?node=%2059%20&id=%20971)<br>anti-FLAG (http://fulaibio.com/index.php?m=home&c=View&a=index&aid=551)<br>anti-p (https://ecmbio.com/products/pp2551)<br>anti-HSP (http://www.ab-mart.com.cn/page.aspx?node=%2089%20&id=%2045223)<br>Goat anti-mouse IgG (H+L)-HRP Conjugated (https://www.amyjet.com/products/115-035-003.shtml)<br>Goat anti-rabbit IgG-HRP Conjugated<br>(http://www.ab-mart.com.cn/page.aspx?node=%2062%20&id=%20980)

Eukaryotic cell lines

Policy information about cell lines and Sex and Gender in Research

Cell line source(s)	Sf21 insect cell line and Hi5 insect cell line (Invitrogen)
Authentication	none of the cell lines were authenticated
Mycoplasma contamination	cell line was not tested for mycoplasma contamination
Commonly misidentified lines<br>(See ICLAC register)	*Name any commonly misidentified cell lines used in the study and provide a rationale for their use.*
